# CD8 malignant proliferation in association with human T cell lymphotropic Virus 1 infection: a case report

**DOI:** 10.1186/1742-4690-12-S1-P68

**Published:** 2015-08-28

**Authors:** Huseini Kagdi, Aileen Rowan, Fiona Child, Edwardo Calonje, Mufaddal Moomin, Maria Antoinietta Demontis, Charles Bangham, Graham Taylor, Paul Fields

**Affiliations:** 1 Section of Retrovirology and GU medicine, Department of Medicine, Imperial College London, London, England, W2 1PG, UK; 2 Section of Virology, Department of Medicine, Imperial College London, London, England, W2 1PG, UK; 3 Department of Dermatology, St John's Institute of Dermatology, London, England, SE1 7EH, UK; 4 Department of Dermatopathology, St John's Institute of Dermatology, London, England, SE1 7EH, UK; 5 Department of Histopathology, Guy's and St Thomas’ Hospitals, Guy's & St Thomas’ Foundation Trust, Westminster Bridge Road, London SE1 7EH, UK; 6 Department of Haematology Guy's and St Thomas’ Hospitals, Guy's & St Thomas’ Foundation Trust, Westminster Bridge Road, London SE1 7EH, UK

## 

Adult T cell leukaemia/lymphoma (ATL) is a predominantly CD4+ T cell neoplasia caused by human T cell lymphotropic Virus 1 (HTLV-1) infection. A few cases of CD8+ ATL have been reported but its existence has been debated. An Afrocaribbean female presented to the dermatology clinic with widespread papulo-nodular rash. HTLV-1 infection was detected by serology. Skin biopsy showed dense dermal infiltration by mainly CD8+ T cells which also expressed CD2, CD3, CD5, CD7 and occasionally CD25. Further investigations showed a mild lymphocytosis (4.4 x 106/L), circulating flower cells, absolute CD4+ count 1247 x 106/L (28.3% of lymphocytes), absolute CD8+ count x 2617 x 106/L (59.8% of lymphocytes), LDH 241 U/L (NR 135 – 214), Calcium 2.7 mmol/L (NR 2.2 – 2.6) but with increased serum PTH level. HTLV-1 proviral load (PVL) in peripheral blood mononuclear cells was 46%. PET/CT scan demonstrated FDG avid lymph nodes above and below the diaphragm. Axillary lymph node core biopsy showed CD20+ B cells nodules admixed with mainly small-to-medium sized T cells and a small population of large T cells. The small lymphocytes were mainly CD8+ T cells (also expressed CD2, CD3, CD5, and CD7 whereas the large cells expressed CD8, CD25 and CD30. Fewer than 5% expressed Ki67 (MIB-1). TCR clonality studies by PCR revealed expansion of an identical clone in all three compartments (skin, lymph node and blood). Detailed immunophenotyping on peripheral blood demonstrated a single dominant TCR vβ1+ clone (>90% of total CD8 T cells) which was CD3+, CD7+, CCR4+, CD25 low and, TSCL-1-. In summary we report a malignant CD8+ clone with atypical immunophenotype in a patient with high PVL. To confirm a working diagnosis of CD8+ ATL high throughput sequencing of each proviral integration sites is underway.

